# Comparing efficacy and safety between Naldebain^**®**^ and intravenous patient-controlled analgesia with fentanyl for pain management post-laparotomy: study protocol for a randomized controlled, non-inferior trial

**DOI:** 10.1186/s13063-019-3260-4

**Published:** 2019-03-18

**Authors:** Hsiang-Lin Tsai, Tsung-Kun Chang, Wei-Chih Su, Yung-Sung Yeh, Ching-Wen Huang, Cheng-Jen Ma, Jaw-Yuan Wang

**Affiliations:** 1 Division of Colorectal Surgery, Department of Surgery, Kaohsiung Medical University Hospital, Kaohsiung Medical University, No. 100 Tzyou 1st Road, San-Ming District, Kaohsiung, 807 Taiwan; 2 Department of Surgery, Faculty of Medicine, College of Medicine, Kaohsiung Medical University, Kaohsiung, Taiwan; 3 Graduate Institute of Clinical Medicine, College of Medicine, Kaohsiung Medical University, Kaohsiung, Taiwan; 4 Division of Trauma and Critical Care, Department of Surgery, Kaohsiung Medical University Hospital, Kaohsiung Medical University, Kaohsiung, Taiwan; 5 Graduate Institute of Medicine, College of Medicine, Kaohsiung Medical University, Kaohsiung, Taiwan; 6 Division of General and Digestive Surgery, Department of Surgery, Kaohsiung Medical University Hospital, Kaohsiung Medical University, Kaohsiung, Taiwan; 7 Center for Biomarkers and Biotech Drugs, Kaohsiung Medical University, Kaohsiung, Taiwan

**Keywords:** Efficacy and safety, Naldebain^®^, Fentanyl, Patient-controlled analgesia, Post-laparotomy

## Abstract

**Background:**

A long-acting prodrug of nalbuphine, nalbuphine sebacate, has been developed for meeting the unmet medical need of long-acting analgesics. Naldebain^®^ (nalbuphine sebacate) has been developed as a new premedication for postoperative pain management. The primary objective of this study is to determine the efficacy and safety of a single dose of intramuscular Naldebain^®^ in patients scheduled to undergo elective laparotomy.

**Methods/design:**

A total of 110 patients will be recruited and randomized into two treatment groups. Group 1 receives a single dose of Naldebain^®^ intramuscularly 24 ± 12 h prior to surgery. Group 2 receives intravenous patient-controlled analgesia (PCA) with fentanyl through 48 h postsurgery. Both groups will have follow-up observations until the final visit (day of discharge, day 6–30). The primary efficacy endpoint is to assess time-specific pain intensity calculated as the area under the curve (AUC) of a visual analog scale at individual time points and by using total AUC. Safety endpoints—including incidence of treatment, emergent adverse events, and percentage of abnormality from baseline to final visit—in vital signs, laboratory tests, and injection site evaluations will also be analyzed. Statistical analyses will be performed on the data to compare the two groups.

**Discussion:**

Post-laparotomy pain can have a harmful effect on patient recovery; therefore, a slow-release formulation that can cover at least 7 days of analgesic effect is required. This study will demonstrate whether a single use of Naldebain^®^ is not less efficacious than PCA with fentanyl for pain management as a non-inferior trial.

**Trial registration:**

NCT03296488.

**Electronic supplementary material:**

The online version of this article (10.1186/s13063-019-3260-4) contains supplementary material, which is available to authorized users.

## Background

### Disease background

Most patients who undergo surgery experience acute postoperative pain; however, evidence suggests that fewer than half of the patients achieve adequate or satisfactory postoperative pain relief [1]. Postoperative pain, especially when poorly managed, leads to harmful acute effects (i.e., adverse physiological responses) and increases the risk of postoperative complications and persistent postoperative pain [2]. Persistent postoperative pain is an intricate response to tissue trauma during surgery that stimulates hypersensitivity of the central nervous system, which causes pain in areas not directly affected by the operative procedure. Either acute or chronic postoperative pain can increase the possibility of postoperative complications, increase the cost of medical care, and complicate wound recovery and subsequent return to normal activities. Pain control following surgery is a major priority for both patients and surgeons.

Several preoperative, intraoperative, and postoperative interventions and management strategies are available for reducing and managing postoperative pain. Opioids and nonsteroidal anti-inflammatory drugs (NSAIDs) are commonly used for postoperative pain management [3]. When given systematically, however, these drugs cause side effects including nausea, vomiting, dizziness, sedation, and pruritus [4]. Opioids can cause respiratory depression, and NSAIDs can impair renal function [5].

### Investigational product

Naldebain^®^ (150 mg nalbuphine sebacate, 75 mg/mL, 2 mL/vial), developed by Lumosa Therapeutic, Inc. (Taipei, Taiwan), is a new premedication for postoperative pain management.

### Rationale

A long-acting prodrug of nalbuphine, nalbuphine sebacate, has been developed for meeting the unmet medical need of long-acting analgesics. Nalbuphine sebacate (Naldebain^®^) is formed by the esterification of two equivalents of nalbuphine with one equivalent of sebacoyl chloride. According to preclinical animal studies, the median lethal dose value of nalbuphine sebacate is considered to be 300 mg/kg for dogs following a single intramuscular administration of nalbuphine sebacate oily solution. The no observed adverse effect level is considered to be less than 30 mg/kg in dogs. In humans, several clinical studies have been conducted to investigate the pharmacokinetics, bioavailability [6–8], efficacy, and safety of Naldebain^®^. The currently proposed clinical use of nalbuphine sebacate is a single dose of Naldebain^®^ administered intramuscularly approximately 24 h prior to the planned surgery for pain relief.

#### Objectives

The primary objective of this study is to determine the safety and efficacy of a single dose of intramuscular Naldebain^®^ administered preoperatively to patients scheduled to undergo elective laparotomy compared with the safety and efficacy of patient-controlled analgesia (PCA) with fentanyl. The study will demonstrate whether a single use of Naldebain^®^ is less efficacious or not than PCA with fentanyl.

For the evaluation of efficacy, the primary endpoint was defined as follows: the area under the curve (AUC) of visual analog scale (VAS) pain intensity scores through 48 h following surgery. The secondary endpoints were as follows: 1) the total consumption (mg) of supplemental analgesics administered postsurgery; 2) pain assessment calculated as the AUC of VAS pain intensity scores from the end of surgery through day 6; 3) pain intensity and interference of the brief pain inventory (BPI); 4) patient satisfaction on a five-point scale; and 5) length of postoperative hospital stay.

For the safety evaluation, two points were considered: 1) the incidence of treatment-emergent adverse events (TEAE); and 2) the abnormality percentage change from baseline to the final visit for vital signs, laboratory tests, and injection site evaluations.

## Methods and design

### Overall design

A randomized, positive-controlled, single-dose, parallel-design study will be conducted with 110 male and female patients scheduled to undergo elective laparotomy to assess the safety and efficacy of an intramuscular injection of Naldebain^®^. The study was designed to include a 5% dropout rate as taken from a previous published work [8].

A total of 110 eligible hospitalized patients will be randomized into one of two treatment groups in a 1:1 ratio. Group 1 is the intramuscular Naldebain^®^ (IM-Naldebain^®^ group) and Group 2 is the intravenous PCA with fentanyl (IV-PCA group). Group 1 receives a single dose of Naldebain^®^ (150 mg nalbuphine sebacate) intramuscularly 24 ± 12 h before surgery. Group 2 receives intravenous PCA with fentanyl through 48 h postsurgery. If PCA with fentanyl is deemed unnecessary by both investigator and patient, the PCA machine can be removed prior to the completion of 48 h as required. If patients require additional medication for treatment of pain, ketorolac and morphine can be used as supplemental analgesics as required. Both groups will undergo follow-up observation until the final visit (from the day of discharge through day 6–30). Statistical analyses will be performed on the data to compare the two groups.

### Number of patients and randomization

Assuming an equivalence margin of 10 and a standard deviation of 18 [8], 104 patients are required to achieve 80% power using the non-inferiority test with a one-sided 97.5% confidence interval (equivalent to a two-sided 95% confidence interval). A total of 110 eligible patients will be randomized in this study, with a predicted dropout rate of 5%. The participants will be randomized using sealed, opaque, individually numbered envelopes, with a restriction of selecting one per person. The envelopes contain data sheets with information on group allocation and the randomization number, generated by the statistician with SAS software. A randomization schedule stratified by center has been prepared. The schedule links sequential numbers to treatment codes allocated randomly. The schedule was prepared with a 1:1 block randomization ratio.

Eligible patients will be randomized to the study medication in accordance with the randomization schedule. The next eligible patient will receive the study medication with the lowest available randomization number. Patients will be only given the study medication carrying their randomization number.

### Study duration

The treatment and observation duration of the study is approximately 8 days. The study consists of a 30-day screening period.

#### Inclusion criteria and exclusion criteria

##### Inclusion criteria

Patients must fulfill all of the following criteria to be eligible for the study: 1) male or female patient between 20 and 80 years of age at screening; 2) scheduled to electively undergo open laparotomy; 3) American Society of Anesthesiology physical class 1–3; and 4) ability and willingness to provide informed consent.

##### Exclusion criteria

Any of the following criteria disqualifies the patient from participation: 1) body mass index (BMI) less than 18 kg/m^2^ or greater than 30 kg/m^2^; 2) history of previous open laparotomy; 3) the planned surgery might cause major complications or requirement of blood transfusion (life-threatened level) based on the medical history and current physical condition of the patient; 4) history of hypersensitivity or adverse reaction to local anesthetics, opioids, or any ingredient of the medications administered in this study; 5) severe comorbidity; 6) chronic preoperative opioid consumption; 7) pregnancy or breastfeeding; and 8) inability to use the PCA device.

### Terminations and withdrawals

Patients are advised that they are free to withdraw from the study at any time. The investigator may also withdraw the patient from the study at any time if, for example, patients become ill or their behavior may compromise the outcome of the study. Any withdrawal of patients from the study will be documented in the final report. The decision to stop treatment will be taken by the investigator.

### Study treatment

#### Description of investigational product and the comparator product

Eligible patients will be randomly assigned to receive one of the following treatments: Group 1—Naldebain^®^ injection (150 mg nalbuphine sebacate, 75 mg/mL, 2 ml/vial); Group 2—PCA with fentanyl (not more than 300 μg/day)

### Study drug administration

Group 1 receives an intramuscular single dose of Naldebain^®^ 24 ± 12 h prior to surgery. The suggested injection site is the musculature of the buttocks. Care is taken to ensure that the drug is not injected into the adipose tissue. Group 2 receives intravenous PCA with fentanyl through 48 h postsurgery.

### Packing and labeling

All drugs used in the study are commercially packed and labeled.

### Handling and storage

Naldebain^®^ is stored in a dry place at a temperature of 2–8 °C and kept away from direct light exposure and excessive humidity. The study investigator or the designated pharmacist is in charge of the management and dispensation of the study medications.

### Product accountability

Drug dispensing documentation is maintained by the delegated investigator, nurse, or pharmacist.

### Treatment compliance

Throughout the study the time of study medication administration is recorded using the case report form (CRF) which is reviewed at all study visits.

### Concomitant therapy

All concomitant medication/therapy being taken by patients upon entry into the study and all treatments given in addition to the study medication during the study are regarded as concomitant treatments and are documented.

### Supplemental analgesics

If patients are unable to achieve satisfactory pain relief from the assigned treatments by randomization, supplemental analgesics may be administered. Ketorolac and morphine may be used as supplemental analgesics as required. All such medication has the start date, stop date (if not ongoing), medication name (generic preferred), dose, frequency, route, and reason for use recorded as part of concomitant therapy.

If patients are hypersensitive to Naldebain^®^, immediate intravenous administration of Narcan^®^ (naloxone hydrochloride) is a specific antidote. Oxygen, intravenous fluids, vasopressors, and other supportive measures are used as indicated.

### Study assessments and procedures (see Table 1)

#### Schedule of assessments

##### Screening (days −30 to −1; all study patients)

The nature of the study, as well as the potential risks and benefits associated with study participation, are fully explained to all potential patients. The following are obtained: 1) informed consent; 2) demographic information, including sex, age, BMI, and type of surgery; 3) vital signs, including temperature, respiratory rate, blood pressure, and heart rate; 4) medical history, including medication use and any history of allergies; 5) physical examination; 6) laboratory testing, including hematological and biochemical tests, and urinalysis; 7) electrocardiogram (ECG) and x-ray; and 8) BPI is evaluated at day –1.

**Table 1:** Study event flowchart

Period	Screening	Study period						
Study day	−30 to −1	−1	0	1	2	3–5	6	Discharge
Informed consent	X							
Medical history	X							
Demographic data	X							
Physical examination	X							X
Vital signs	X	X	X	X	X	X	X	X^d^
Injection site evaluation		X^b^	X^b^	X^b^	X^b^	X^b^	X^b^	X^b,d^
Laboratory tests	X	X^a^						X
12-lead ECG/chest x-ray	X							
Inclusion/exclusion criteria		X						
Randomization		X						
Naldebain^®^ administration		X^b^						
Surgery			X					
Pain assessment		X	X	X	X	X	X	
BPI		X			X		X	
Patient satisfaction								X
Concomitant medication	X	X	X	X	X	X	X	X^d^
Adverse event record		X	X	X	X	X	X	X^d^
Drug accountability for fentanyl			X^e^	X^e^	X^e^			
Discharge								X^c^
End of study								X

*BPI* brief pain inventory, *ECG* electrocardiogram

^a^Additional laboratory tests are requested as required by the investigator

^b^Only for subjects who are randomized to the Naldebain^®^ group

^c^Patients can discharge on day 6–30; patients still hospitalized after day 6 do not serve as a protocol deviation/violation or serious adverse event

^d^For patients who are discharged on day 6, the assessment is performed only once

^e^Only for subjects who are randomized to the patient-controlled analgesia (PCA) group

**Fig. 1:**
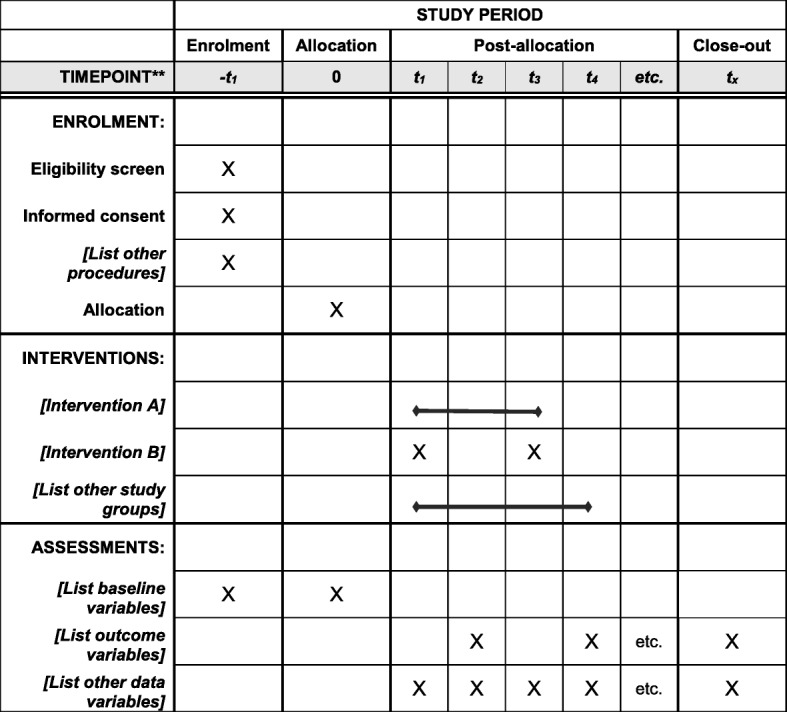
Example template of recommended content for the schedule of enrolment, interventions, and assessments

##### Study day 1 (all eligible patients)

Eligible patients are required to check into the clinical site before surgery (day 0). The following procedures are performed at check-in: 1) review inclusion/exclusion criteria; 2) randomization; 3) review of concomitant medications; and 4) review of adverse events (AEs).

Additional laboratory tests are requested as required by the investigator.

All eligible patients are hospitalized and randomized into the two treatment groups: Group 1—intramuscular Naldebain^®^ (150 mg nalbuphine sebacate) 24 ± 12 h before surgery; Group 2—Intravenous PCA with fentanyl through 48 h postsurgery.

For pain assessment, patients in Groups 1 and 2 rate their pain intensity using the VAS pain scale prior to dosing or at day 1, respectively.

Vital signs are checked prior to dosing and once daily until day 6.

Group 1 receives Naldebain^®^, and the injection site is evaluated within 1 h prior to dosing.

##### Study day 0 to 5

All patients are given general anesthesia prior to their scheduled surgical procedure. If patients require additional medication for the treatment of pain, ketorolac and morphine can be used as supplemental analgesics as required. Post-surgery, the following evaluations are performed: 1) for pain assessment, all patients rate their average pain intensity using a VAS pain scale (assessments are performed before the first use of PCA or supplemental analgesics and then at 4 ± 1, 24 ± 2, 32 ± 3, and 48 ± 4 h, and once daily on day 3 to day 5 following surgery); 2) BPI evaluation is performed on day 2; 3) vital signs (temperature, respiratory rate, blood pressure, and heart rate) are examined once daily prior to the final visit; 4) in Group 1 (the IM-Naldebain^®^ group), the injection site is evaluated once daily until the final visit; 5) a review of concomitant medication and adverse events are record; and 6) in Group 2 (the IV-PCA group), the amount of fentanyl used is recorded from day 0 through day 2.

##### Study day 6

The following evaluations are performed at day 6: 1) for pain assessment, patients rate their pain intensity in the patient diary; 2) BPI is evaluated; 3) vital signs (temperature, respiratory rate, blood pressure and heart rate) are examined; 4) the injection site is evaluated; 5) a review of the AE record; and 6) a review of concomitant medication.

##### Final visit (discharge, day 6–30)

For safety concerns, the study will follow-up each patient until discharge (within a time window for discharge of 6–30 days). The patients will be evaluated for: 1) vital sign (temperature, respiratory rate, blood pressure, and heart rate) examination; 2) injection site evaluation; 3) laboratory tests, including hematological and biochemical tests, and urinalysis; 4) patient satisfaction (at the final visit, each patient is asked the following question (Table 2): “How satisfied were you with your postsurgical analgesia?” and patients are asked to classify themselves as either “highly satisfied,” “satisfied,” “uncertain,” “dissatisfied,” or “very dissatisfied”); 5) physical examination; 6) review of the AE record; and 7) review of concomitant medication.

**Table 2:** Five-point rating of patient satisfaction

Rating	Description
1	Highly satisfied
2	Satisfied
3	Uncertain
4	Dissatisfied
5	Very dissatisfied

#### Patient background

The following items are investigated at the screening visit and the results are recorded on the CRF for the patient background data: informed consent, demographic information, vital signs, medical history, physical examination, 12-lead ECG, chest x-ray, and laboratory testing (complete blood count with differential, platelets, biochemical tests, and urinalysis).

#### VAS and patient diary

The VAS is a 10-cm numeric scale (0 = no pain to 10 = worst pain ever).

#### Short-form BPI

The short-form BPI is a self-reported scale that measures the severity of pain based on average pain (severity scores 0 = no pain to 10 = most severe pain). BPI is evaluated at days −1, 2, and 6.

#### Injection site evaluation (Table 3)

The injection site reaction is evaluated prior to dosing and once daily from day 1 to the final visit. A five-point rating is used (0 = no reaction, 1 = no swelling or erythema present, 2 = erythema at the site, no swelling is present, 3 = erythema and swelling present at the site, no treatment required, 4 = erythema and swelling present, treatment is required).

**Table 3:** Five-point rating grade when assessing the injection site

Grade	Definition
0	No reaction
1	Burning/stinging or pain at the site of injection; no swelling or erythema present
2	Erythema at the site; no swelling present
3	Erythema and swelling present at the site; no treatment required
4	Erythema and swelling present; treatment required

#### Adverse events

An AE is any untoward medical occurrence in a patient administered the study drug that does not necessarily have a causal relationship with the treatment. An AE can be any unfavorable and unintended sign, symptom, or disease temporally associated with the use of the study drug, regardless of whether it is considered to be related to the study drug. This includes any newly occurring event or previous condition that has increased in intensity or frequency since the administration of the study drug. Lack of efficacy before the study end is not considered an AE.

A serious AE (SAE) is defined as an AE that meets any of the following criteria (Table 4): 1) results in death; 2) is life threatening; 3) results in inpatient hospitalization or prolongation of existing hospitalization; 4) results in a persistent or significant disability/incapacity; 5) results in congenital anomaly/birth defect; and 6) an important medical event.

**Table 4:** Severity categories of adverse events

Severity	Description
Mild	Event may be noticeable to patient; does not influence daily activities; usually does not require intervention
Moderate	Event may be of sufficient severity to make patient uncomfortable; performance of daily activities may be influenced; intervention may be required
Severe	Event may cause severe discomfort; usually interferes with daily activities; patient may not be able to continue in the study; treatment or other intervention usually required
Serious	Results in death or life-threatening situation; inpatient hospitalization or prolongation of existing hospitalization; persistent or significant disability/incapacity; or congenital anomaly/birth defect or important medical event

The investigator evaluates the intensity of each AE using the following definitions (Table 4):

Mild—event may be noticeable to the patient; does not influence daily activities; usually does not require intervention.

Moderate—event may be of sufficient severity to make patient uncomfortable; performance of daily activities may be influenced; intervention may be required.

Severe—event may cause severe discomfort; usually interferes with daily activities; patient may not be able to continue in the study; treatment or other intervention usually required.

After careful consideration, the investigator evaluates the relationship of each AE to the study drug by applying the following definitions (Table 5):

**Table 5:** Determining the relationship between adverse events and the study drug

Relationship	Description
Certain	An adverse event, including laboratory test abnormality, occurring in a plausible time relationship to drug administration, which cannot be explained by concurrent disease or other drugs or chemicals
Probable/likely	An adverse event, including laboratory test abnormality, with a reasonable time sequence to administration of the drug that is unlikely to be attributed to a plausible response on withdrawal
Possible	An adverse event, including laboratory test abnormality, with a reasonable time sequence to administration of the drug, which could also be explained by concurrent disease or other drugs or chemicals. Information on drug withdrawal may be lacking or unclear
Unlikely	An adverse event, including laboratory test abnormality, with a temporal relationship to drug administration which makes a causal relationship improbable, in which other drugs, chemicals, or underlying disease provide plausible explanations
Not related	An adverse event which is judged to be clearly due to extraneous causes (e.g., disease and environment)

Certain—an AE, including laboratory test abnormality, occurring in a plausible time relationship to drug administration, which cannot be explained by concurrent disease or other drugs or chemicals. Response to withdrawal of the drug (de-challenge) should be clinically plausible. The event must be definitive pharmacologically or phenomenologically, using a satisfactory re-challenge procedure if necessary.

Probable/likely—an AE, including laboratory test abnormality, with a reasonable time sequence to administration of the drug that is unlikely to be attributed to a plausible response on withdrawal. Re-challenge information is not required to fulfill this definition.

Possible—an AE, including laboratory test abnormality, with a reasonable time sequence to administration of the drug, but which could also be explained by concurrent disease or other drugs or chemicals. Information on drug withdrawal may be lacking or unclear.

Unlikely—an AE, including laboratory test abnormality, with a temporal relationship to administration of the drug which makes a causal relationship improbable, and in which other drugs, chemicals, or underlying disease provide plausible explanations.

Not related—an AE that is judged to be clearly due to extraneous causes (e.g., disease and environment).

#### Adverse event reporting

All AEs, regardless of causality, that occur between the time the study medication is administered and completion of the treatment phase are reported. Those meeting the definition of SAEs are reported using the SAE form.

All AEs, regardless of seriousness, severity, or presumed relationship to the study drug, are recorded using medical terminology in the source document and the CRF. Whenever possible, diagnoses are given when signs and symptoms are due to a common etiology (e.g., cough, runny nose, sneezing, sore throat, and head congestion should be reported as “upper respiratory infection”). Investigators must record in the CRF their opinion concerning the relationship of the AE to study therapy. All measures required for AE management must be recorded in the source document and according to sponsor instructions.

The investigator must report all such events to the appropriate independent ethics committee (IEC)/institution review board (IRB) that approved the protocol unless otherwise required and documented by the IEC/IRB.

#### Serious adverse events

Information regarding SAEs is transmitted to the sponsor using the SAE form, which must be signed by a member of the investigational staff.

All SAEs that have not been resolved by the end of the study, or that have not resolved upon discontinuation of the patient’s participation in the study, must be followed up until any of the following occurs: 1) the event resolves; 2) the event stabilizes; 3) the event returns to baseline, if a baseline value is available; 4) the event can be attributed to agents other than the study drug or to factors unrelated to study conduct; and 5) when it becomes unlikely that any additional information can be obtained (patient or healthcare practitioner refusal to provide additional information, lost to follow-up after demonstration of due diligence with follow-up efforts).

The death of a patient in a clinical study, regardless of whether the event is exempted from association with the investigational agent, is considered an SAE.

### Data management and statistical methods

#### Study endpoints

The following endpoints will be evaluated.

The primary efficacy endpoint is the pain assessment (time-specific pain intensity) calculated as the AUC of VAS pain intensity scores through 48 h postsurgery.

The secondary efficacy endpoints are as follows: 1) the total consumption (mg) of supplemental analgesics administered postsurgery; 2) pain assessment calculated as the AUC of VAS pain intensity scores from the end of surgery through day 6; 3) pain intensity and interference of BPI; 4) patient satisfaction on a five-point rating (Table 2); and 5) length of postoperative hospital stay.

The safety endpoints are as follows: 1) incidence of TEAE; and 2) percentage of abnormality from baseline to final visit in vital signs, laboratory tests, and injection site evaluations.

#### Analysis populations

Efficacy, safety, and general characteristics data will be analyzed for all randomized patients. Primary efficacy analysis will also be conducted on the per-protocol (PP) population. The primary endpoint will analyze all randomized patients (on an intent-to-treat (ITT) basis) and per-protocol (PP) population.

The ITT population is defined as all randomized patients. The PP population is defined as all randomized patients without major protocol violations.

#### Hypotheses and level of significance

Statistical analyses will be performed on the data to compare the Naldebain^®^ group with the PCA group. All statistical tests will be two-sided and evaluated at a level of significance of 0.05.

#### Dropouts, premature termination, and missing values

The dropouts, premature termination of study medication, and withdrawal will be summarized and analyzed according to treatment groups. A last observation carried forward procedure will be used to estimate the missing data for efficacy variables.

#### Baseline demographics

Descriptive statistics for demographic and background information are obtained for both groups at the screening visit.

#### Safety analysis

For the safety analysis, continuous variables will be tested using a *t* test and categorical variables will be compared by chi-square or Fisher’s exact test. The coding system used will be the Medical Dictionary for Regulatory Activities (MedDRA).

AEs will be summarized descriptively according to body system, MedDRA preferred term, center, treatment, and overall outcome. If more than one type of event occurs within a system organ class/preferred term for the subject, the subject is counted only once when summarizing the data by system organ class/preferred term. Incidence tables of intensity, drug relationship, drug-related AE (including certain, probable, and possible), action taken regarding the study drug, and whether treatment was required for an event or SAE will be presented by center, treatment, and overall outcome. Incidence rates ≧2%, ≧10%, and rates for Naldebain^®^ greater than two times PCA fentanyl will be presented.

In terms of severity and relationship summaries, the most severe event and the most directly related event will be presented in summary tables in the case of multiple occurrences per subject. Only treatment-emergent events will be included in the AE summary; other events will only be listed. Treatment-emergent events include events that start on or after the first day of study drug administration, that were not present at baseline, or were present at baseline but increased in severity after the start of study drug administration.

A frequency table of injection site will be presented according to center, time, and treatment. Descriptive statistics of recovery duration (hours, defined as from first score 0 over second score 0 or 1) for injection site assessment will be presented according to center and treatment.

Incidence of SAEs will be tabulated. SAE incidents will be summarized descriptively according to center, system organ class, preferred term, treatment group, and overall outcome.

For laboratory data, descriptive statistics of hematology, biochemistry, and urinalysis data will be presented according to parameter, center, visit, treatment, and overall data. Net changes from baseline (treatment–baseline) of hematology, biochemistry, and urinalysis results will be compared between treatments using analysis of variation (ANOVA) with baseline as the covariate. The baseline for laboratory data will be defined as the screening visit. If no data exist for the screening visit, the baseline will be defined as day 0.

For vital signs, descriptive statistics of vital signs including systolic and diastolic blood pressure, heart rate, respiratory rate, and body temperature will be presented according to center, visit, treatment, and overall outcome. Net change of vital signs compared with baseline (treatment–baseline) will be analyzed between treatments using ANOVA with baseline as the covariate. A frequency table of physical examinations with abnormal results will also be presented according to center, treatment, and overall outcome.

#### Efficacy analysis

Pain intensity will be analyzed at individual time points and by AUC. The mean AUCs at 0–24 h and 0–48 h for Naldebain^®^ and PCA will be calculated using the trapezoidal method. These values will then be compared using a two-sample *t* test.

For total postsurgical consumption of supplemental analgesics, opioid and NSAIDs will be counted separately. A two-sample *t* test will be used to compare treatment groups. Between-group differences at baseline in answers to BPI questions based on rating scales will be analyzed using a two-sample *t* test; an analysis of covariance model will then be used for postsurgical BPI assessments with baseline response as the covariate. A chi-square test will be used to compare the Naldebain^®^ and PCA groups with respect to patient satisfaction with postsurgical analgesia. Length of postoperative hospital stay will be tested using a two-sample *t* test.

#### Interim analysis

No interim analysis will be performed.

### Ethical and regulatory aspects

#### Good clinical practice

This study will be conducted in compliance with the following documents: 1) the International Conference on Harmonization (ICH) Harmonized Tripartite Guidelines for Good Clinical Practice (GCP) 1996; 2) the Taiwan Good Clinical Practice, January 6, 2005; and 3) the Declaration of Helsinki.

#### Institutional review board approval

The study is being conducted under the supervision of an IRB/IEC. ICH guidelines require studies to have obtained approval from an IRB (KMUHIRB-F(I)-20,170,089) prior to the enrollment of human subjects into a study.

#### Delegation of investigator responsibilities

All investigators are responsible for the conduct of the trial in accordance with the protocol. In addition, the responsibilities of an investigator extend to the following: 1) providing sponsors with written documentation that the study protocol, any protocol amendments, and informed consent forms have received IRB/IEC approval; 2) reporting to the IRB/EC as required (the IRB/EC must assume continued responsibility for the study and review the research on an annual basis); 3) maintaining a file of all communications with the IRB/EC on issues related to the clinical trial; and 4) conducting the study according to the protocol, ICH–GCP guidelines, and in accordance with the Declaration of Helsinki.

#### Informed consent

Written informed consent must be obtained from all patients prior to study participation.

#### Confidentiality

Only the patient number and patient initials are recorded in the CRF. The investigator maintains a personal patient identification list (patient numbers with the corresponding patient names) to enable records to be identified.

## Discussion

Approximately 35 million surgical procedures are performed as inpatient procedures annually in the United States [9, 10]. Postoperative pain is common, and pain intensity can be moderate to severe depending on the surgical site [11, 12] in the first few days postsurgery [13]. An estimated 15% to 45% of patients experience chronic postoperative pain [14, 15]. When controlled poorly, postoperative pain can have a significant effect on patient recovery. Over half of postoperative patients experience inadequate pain relief. Consequently, a slow-release formulation that can cover at least 7 days of analgesic effect is required. Proper management of postoperative pain is necessary to relieve pain and hasten mobilization, shorten hospital stays, reduce hospital costs, and increase patient satisfaction [16].

Pain is the natural response of the body to tissue injury, and both the injury itself and the following inflammatory reaction near the injured site contribute to pain. Uncontrolled postoperative pain may activate the sympathetic nervous system, which may increase myocardial oxygen consumption and increase the risk of myocardial ischemia or infarction. Sympathetic activation may also reduce gastrointestinal motility, which may result in postoperative ileus. Local anesthetics act as sodium channel blockers on nerve fibers, inhibiting the conduction of nerve impulses from the target site to the central nervous system. Long-acting local anesthetics can be employed for postoperative pain control owing to their prolonged anesthetic effect. However, the longest duration of analgesic effect in one single injection is still limited to only 4–6 h, and this is believed to restrict the use of local anesthetics for pain control in major surgical procedures such as laparotomy.

Open abdominal surgery is associated with postoperative pain, nausea, ileus, and prolonged hospital stay with associated costs [17]. Although opioids have been the mainstay of perioperative analgesia, they are significantly associated with postoperative ileus, especially when daily dosing exceeds 2 mg of intravenous hydromorphone equivalents [18]. Fentanyl-based intravenous PCA (IV-PCA) requires a higher dose of opioids to acquire satisfactory analgesic effects. This, in turn, produces adverse effects such as nausea, vomiting, and pruritus, which causes patients to discontinue the use of IV-PCA [19, 20]. Nalbuphine is a semisynthetic opioid indicated for the relief of moderate to severe pain. Its short half-life requires frequent injections in clinical practice, resulting in a greater incidence of AEs. A prodrug of nalbuphine, nalbuphine sebacate, has been developed, dissolved in a simple oil-based injectable formulation that can deliver and maintain an effective blood level of nalbuphine [6]. Tien et al. demonstrated that the complete release of nalbuphine sebacate into the blood stream required approximately 6 days, during which nalbuphine sebacate was rapidly hydrolyzed to nalbuphine; this suggests that a single injection of 150 mg nalbuphine sebacate can provide long-lasting pain relief [6].

## Trial status

The trial began in September 2017 and is expected to finish in December 2018. The first patient was enrolled in October 2017 and there are currently 20 patients, including 10 patients in Group 1 (IM-Naldebain^®^) and 10 patients in Group 2 (IV-PCA with fentanyl), enrolled in the study until the end of January 2018. Importantly, the SPIRIT checklist [21] was referenced in this protocol (Additional file 1) and (Figure 1).

## Additional file

Additional file 1: SPIRIT 2013 checklist: recommended items to address in a clinical trial protocol and related documents. (PDF 107 kb)

## Abbreviations

AE: Adverse event
AUC: Area under the curve
BMI: Body mass index
BPI: Brief pain inventory
CRF: Case report form
ECG: Electrocardiogram
GCP: Good Clinical Practice
ICH: International Conference on Harmonization
IEC: Independent ethics committee
IRB: Institutional review board
MedDRA: Medical dictionary for regulatory activities
NSAID: Nonsteroidal anti-inflammatory drug
PCA: Patient-controlled analgesia
PP: Per-protocol
SAE: Serious adverse event
VAS: Visual analog scale
